# Attitudes Toward General Vaccination Mediate the Association Between Influenza Vaccination History and Pneumococcal Vaccination Intention Among Older Adults in China

**DOI:** 10.3390/vaccines13010020

**Published:** 2024-12-29

**Authors:** Siwen Huang, Chi Ruan, Yan Jiang, Yudan Song, Yuhang Zhang, Zeying Qin, Xinyu Shi, Mengyue Zhang, Jingtao Zhou, Dingwan Chen, Yongkang Xiao, Liang Wang, Lili Tian, Rui Peng, Zongchao Peng, Sitong Luo

**Affiliations:** 1 Vanke School of Public Health, Tsinghua University, Beijing 100084, China; siwen@link.cuhk.edu.hk (S.H.); rc23@mails.tsinghua.edu.cn (C.R.); yuhang-z23@mails.tsinghua.edu.cn (Y.Z.); qinzeying@mail.tsinghua.edu.cn (Z.Q.); shixinyu23@mails.tsinghua.edu.cn (X.S.); kassiezhang@link.cuhk.edu.hk (M.Z.); zhoujt@mail.tsinghua.edu.cn (J.Z.); 2 School of Public Health, Xiangnan University, Chenzhou 423043, China; jyan89@126.com; 3 National Immunization Program, Chinese Center for Disease Control and Prevention, Beijing 100050, China; songyd@chinacdc.cn; 4 School of Public Health, Hangzhou Medical College, Hangzhou 310053, China; chendw@hmc.edu.cn; 5 Department of Acute Infectious Diseases Control and Prevention, Anhui Provincial Center for Disease Control and Prevention, Hefei 230601, China; xiao84791@sina.com; 6 Department of Infectious Diseases Control and Prevention, Chengdu Center for Disease Control and Prevention, Chengdu 610041, China; wangliang@cdcdc.org; 7 Beijing Miyun District Center for Disease Control and Prevention, Beijing 101520, China; guojibingduxue@126.com; 8 School of Government and Public Affairs, Communication University of China, Beijing 100024, China; ruipeng@cuc.edu.cn; 9 School of Public Policy and Management, Tsinghua University, Beijing 100084, China; pengzch@tsinghua.edu.cn; 10 Institute for Healthy China, Tsinghua University, Beijing 100084, China

**Keywords:** vaccination, behavioral spillover, anti-vaccine attitudes, older adults

## Abstract

**Background:** Influenza and pneumococcal vaccinations play a crucial role in disease prevention among older adults and are recommended to older adults aged 60 years and over in China, but the vaccination rates are suboptimal. Behavioral spillover indicates that a change in one behavior may lead to changes in other related behaviors. **Objective:** Based on the Behavioral Spillover Theory, this study aimed to investigate the association between influenza vaccination history and pneumococcal vaccination intention, as well as the mediating role of negative attitudes toward general vaccination among older adults in China. **Method:** A multi-center cross-sectional survey was conducted among 1031 older adults, and 658 participants (median age: 65.0 ± 9.0 years) who had not received pneumococcal vaccination were included in the analysis. Correlation analysis and path analysis were performed. **Results:** A significant positive association was observed between influenza vaccination history and pneumococcal vaccination intention (r = 0.167, *p* < 0.001). In contrast, negative attitudes toward general vaccination, including mistrust of vaccine benefits (r = −0.253, *p* < 0.001), worries about unforeseen future effects (r = −0.180, *p* < 0.001), concerns about commercial profiteering (r = −0.360, *p* < 0.001), and a preference for natural immunity (r = −0.212, *p* < 0.001) were negatively associated with pneumococcal vaccination intention. Negative attitudes toward general vaccination mediated the association between influenza vaccination history and pneumococcal vaccination intention (total indirect effect = 0.119, *p* < 0.001, effect size = 50.0%). **Conclusion:** These findings demonstrated that influenza vaccination history may reduce negative attitudes toward general vaccination, which may further increase pneumococcal vaccination intention, indicating spillover effects of influenza vaccination history. To promote vaccination behavior among older adults, addressing negative attitudes toward general vaccination is crucial.

## 1. Introduction

Populations worldwide are aging at an accelerated pace, with both the number and proportion of people aged 60 years and over increasing rapidly [1]. The World Health Organization (WHO) reports that the global population of individuals aged 60 years and over is projected to reach 1.4 billion by 2030, and it is expected to rise to 2.1 billion by 2050 [2]. In China, this trend is particularly prominent, with approximately 297 million older adults in 2023, accounting for one-fifth of the global elderly population [3,4]. The rapid aging of the population presents significant social challenges, especially health-related issues among the elderly, such as substantial disease burden led by the high prevalence of infectious diseases [5]. Influenza and pneumococcal infection pose a significant threat to the health of older individuals, while vaccination has been proven as an effective strategy to prevent these two diseases. Previous studies reported that influenza vaccines could prevent 50% of infections and the pneumococcal vaccine was 28% to 54% more effective in preventing invasive pneumococcal disease in the elderly [6,7,8]. Meanwhile, the dual vaccination of influenza and pneumococcal vaccines has demonstrated effectiveness rates of 29% for preventing pneumonia, 38% for reducing mortality, 35% for preventing influenza, and 18% for reducing hospitalization [9]. In China, to protect this vulnerable population and promote healthy aging, the government recommends that adults aged 60 years and over should receive influenza and pneumococcal vaccinations [6,10].

However, vaccine hesitancy is prevalent among older adults in China, resulting in suboptimal vaccination rates [10,11]. Vaccine hesitancy, defined as the refusal, hesitation, or delay in getting vaccinated despite the availability of vaccination services, was identified by the WHO as one of the top ten global health threats in 2019 [6]. Notably, psychosocial factors, including negative attitudes toward general vaccination, have increasingly been recognized as significant contributors to vaccine hesitancy, which leads to insufficient vaccination uptake [12]. A growing body of literature documented that individuals’ concerns about vaccine efficacy, fear of side effects, and concerns about profiteering were negatively associated with pneumococcal vaccination intentions or uptake behaviors [13,14,15]. Behavioral spillover is the phenomenon when a behavior change is accompanied by subsequent changes in other behaviors related to the same goal (e.g., protecting health) [16]. Based on the Behavioral Spillover Theory, influenza vaccination history may influence the intention or actual behavior of receiving pneumococcal vaccination, as both are aimed at protecting health [17,18]. Prior empirical studies also found similar associations. A study conducted in Germany found that having received the influenza vaccination was associated with an increased likelihood of receiving the pneumococcal vaccination [19]. Likewise, a study in Australia showed that among adults aged 65 and over, influenza vaccination in the previous 12 months was associated with self-reported pneumococcal vaccination behaviors [20]. Similar results were observed in the U.S., Greece, and Canada [21,22,23]. However, existing studies have only considered the association between one vaccination behavior and the other, but they have not explored the mediation mechanisms underlying the association. Moreover, all of the relevant studies were conducted in other countries or other age groups, but not with older Chinese people.

Considering the impact led by attitudes toward vaccines on vaccination behavior, the influence of influenza vaccination history on pneumococcal vaccination intention might be mediated by negative attitudes toward general vaccination. Positive experiences with vaccinations may reinforce the belief that vaccines are safe and effective in disease prevention, thus reducing negative attitudes toward general vaccination. This shift in perception may, in turn, enhance future vaccination intentions [24]. While several studies have explored the spillover effect of the COVID-19 vaccination behaviors or vaccine hesitancy on general vaccine skepticism and influenza vaccination intentions, studies that investigated the spillover effects of routine vaccines (i.e., influenza vaccine) on negative attitudes toward vaccines and vaccination intention are sparse [25,26]. To the best of our knowledge, only one study examined factors associated with negative attitudes toward vaccination in general and found that prior vaccination against seasonal flu is an important factor [27]. Hence, to improve the vaccination among the elderly, it is crucial to investigate the association between influenza vaccination history and pneumococcal vaccination intention, and the influence of negative attitudes toward the general vaccination.

This secondary analysis of a nationwide cross-sectional study aimed to investigate the association between influenza vaccination history and pneumococcal vaccination intention, as well as the mediating role of negative attitudes toward general vaccination among older adults in China. It is hypothesized that influenza vaccination history may increase pneumococcal vaccination intention, with this association potentially mediated by attitudes toward vaccination.

## 2. Methods

### 2.1. Study Design

The data were taken from a large-scale cross-sectional survey conducted among 1031 older adults in five provincial regions in China from 18 January to 6 February 2024. These regions were selected based on their geographical and economic representativeness, including Beijing (North of China, GDP per capita in 2023: CNY 200,000), Zhejiang (East of China, CNY 125,043), Anhui (Central of China, CNY 76,830), Hunan (South of China, CNY 75,938), and Sichuan (West of China, CNY 71,835) [28]. The study was approved by the Institutional Review Board of Tsinghua University, China (reference number: 20240007).

### 2.2. Sample Size Planning

This is a secondary analysis of a large-scale cross-sectional survey with a sample size of 1031. Considering the purpose of the survey and the available human and financial resources, we planned to recruit at least 200 older adults in each of the five study sites, yielding a total minimum sample size of 1000. Assuming a type I error of 0.05 and a statistical power of 0.8, the sample size of 1000 could detect a minimum effect size (Cohen’s d) of 0.09. In this secondary analysis with a sample size of 658, assuming a type I error of 0.05 and a statistical power of 0.8, the sample size of 658 could detect a minimum effect size (Cohen’s d) of 0.11. The PASS software 11.0 was used to calculate the sample size.

### 2.3. Participant Recruitment

The inclusion criteria of participants were as follows: (a) an age of 60 years or older; (b) eligibility for influenza, pneumococcal, and herpes zoster vaccines; and (c) the provision of informed consent. Older adults diagnosed with psychiatric disorders were excluded. Participants were enrolled using facility-based sampling. Facility-based sampling refers to recruiting members of the target population from various facilities. Staff from the local health commission in each provincial region, after receiving formal training, assisted in screening and inviting potentially eligible individuals to participate in the study. Interested individuals were then screened for eligibility and provided with detailed study information after offering informed consent.

### 2.4. Data Collection

Informed consent was obtained before any data collection. Individuals who agreed to participate were then provided with a QR code to access the self-administered online questionnaire via the Wenjuanxing survey platform (www.wjx.com). Completing the questionnaire took approximately 10 min for each participant. Participants who were unable to complete the electronic questionnaire independently would receive assistance from staff or family members. To minimize the risk of staff or family members influencing participants’ responses, they were instructed to read the questions exactly as written and to select the corresponding options based on the participants’ answers, without offering any suggestions. Eligible participants who completed questionnaires and passed a quality check were compensated with CNY 2–50 via the platform (approximately USD 0.3–6.9) for their time and effort. The quality check of the questionnaire includes the following: (1) an Attention Check Question and (2) the time spent on completing the questionnaire. These criteria were used collectively to evaluate the validity of the questionnaires. The compensation amount was randomly determined through the lottery and participants were entered into the lottery after completing the questionnaire.

**Figure 1:**
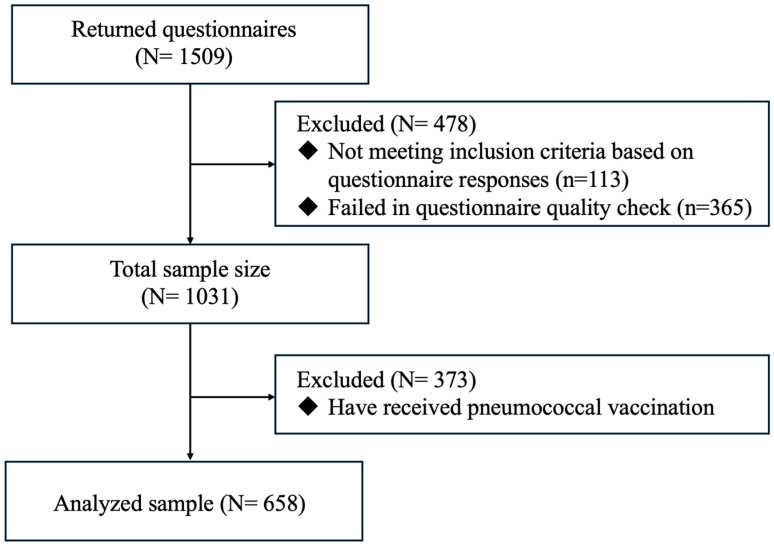
Flowchart of participant selection for the analysis.

A total of 1509 participants completed the questionnaire and 478 participants were subsequently excluded (113 participants did not meet the inclusion criteria based on questionnaire responses and 365 participants failed in the questionnaire quality check) and the total sample size was 1031. Of the 1031 participants, 658 older adults who had not received pneumococcal vaccination were included in the analysis, while 373 individuals who had received pneumococcal vaccination were excluded as they were not eligible for further vaccination within the recommended interval (Figure 1).

### 2.5. Measures

#### 2.5.1. Influenza Vaccination History (Independent Variable)

A single binary item was used to measure the recent uptake of influenza vaccination (“Have you received the influenza vaccine in the past 12 months?”).

#### 2.5.2. Negative Attitudes Toward General Vaccination (Mediators)

In this study, participants were asked to focus on vaccines in general rather than specifically on a vaccine for pneumococcal. The 12-item Vaccination Attitudes Examination Scale (VAX) was used to measure the negative attitudes toward general vaccination. It included the following four subscales: mistrust of vaccine benefit (3-item), worries over unforeseen future effects (3-item), concerns about commercial profiteering (3-item), and preference for natural immunity (3-item) [29]. A sample item was “Vaccines generate significant profits for pharmaceutical companies but provide little benefit to the general public” (ranging from 1 = strongly disagree to 5 = strongly agree). The item scores were summed up, with higher scores indicating more negative attitudes toward general vaccination (Cronbach’s alpha = 0.85). The Chinese version of this scale was used to examine predictors of COVID-19 booster dose uptake and potential moderators (Cronbach’s alphas = 0.81) [30]. Adequate convergent validity and internal reliability (Cronbach’s alphas = 0.66–0.93) were established for all four subscales. Based on a study conducted by Bhatnagar et al., Cronbach’s alpha of 0.65 is acceptable [31].

#### 2.5.3. Pneumococcal Vaccination Intention (Dependent Variable)

First, participants were asked to report whether they had heard of the influenza, pneumococcal, and herpes zoster vaccines. Next, a standardized introduction about pneumococcal vaccines was provided before participants rated their likelihood of receiving the pneumococcal vaccination (response categories: “1 = very unlikely” to “5 = very likely”). A binary variable was created by recoding: “very unlikely” and “unlikely” were recoded as unwilling to receive the pneumococcal vaccine, while “very likely”, “likely”, and “neutral” were recoded as intending to receive the pneumococcal vaccine.

#### 2.5.4. Background Information

Participants’ background information, including their demographic and medical characteristics, was collected. Demographic information included age, gender, ethnicity, education level, marital status, employment status, monthly household income, and living arrangement, while medical characteristics included medical insurance status and chronic disease status.

### 2.6. Statistical Analysis

First, the distributions of all the studied variables were described by medians and interquartile ranges (IQRs) for continuous variables that did not follow normal distribution or frequencies and proportions for categorical variables. To compare the differences in background variables, vaccination-related behaviors, intentions, and perceptions across different regions, chi-square tests or Fisher’s exact tests were used for categorical data, while Kruskal–Wallis H tests were applied to continuous data that were not normally distributed. Second, univariate logistic regression analyses were conducted to explore associations between background variables and pneumococcal vaccination intention, with odds ratios (ORs) and 95% confidence intervals (CIs) reported. After controlling for all background variables (e.g., medical insurance status), multivariable logistic regression analyses were conducted to assess the adjusted associations, with adjusted odds ratios (AOR) and 95% CI values reported. To reduce information loss, age and negative attitudes toward general vaccination were included as continuous variables in the regression and subsequent analyses, whereas other background variables were recoded as binary variables. Third, the Spearman correlation was conducted to test the associations between the main studied variables. Fourth, path analysis was conducted using weighted least squares with mean and variance (WLSMV) estimation to test the hypothesized mediation model after controlling for all background variables. The 95% bias-corrected confidence intervals of the indirect effects were estimated based on 5000 bootstrapped samples. Standardized estimates and the bootstrapped 95% CI values were reported. The analyses were conducted by the SPSS 26.0 (New York, NY, USA) and MPlus 8.3 software (Muthén & Muthén, Los Angeles, CA, USA).

## 3. Results

### 3.1. Background Information, Vaccination-Related Behavior, Intention, and Perceptions

Participants’ background characteristics are summarized in Table 1. Except for gender, marital status, and living status, the background information, vaccination-related behavior, intention, and perceptions were significantly different in each region. Among the 658 participants, the median age was 65.0 years (IQR = 9.0). The majority were female (n = 350, 53.2%), married or living with a partner (n = 569, 86.5%), and had an education level of high school or below (n = 607, 92.2%). More than half of the participants were retired (n = 398, 60.5%), and nearly half reported a monthly household income of CNY ≤3000 (n = 290, 44.1%). Most participants lived with a spouse (n = 493, 74.9%), had medical insurance (n = 558, 84.8%), and 78.6% reported having at least one chronic disease.

Regarding vaccination-related information, 55.2% of participants had received the influenza vaccine in the past 12 months. More than half of the participants had not heard of pneumococcal vaccination before (n = 438, 66.6%). Pneumococcal vaccination intention was relatively low, with 10.3% indicating they were “very unlikely” to vaccinate and only 11.7% indicating they were “very likely” to vaccinate. Negative attitudes toward general vaccination were prevalent, with a median VAX score of 28.5 (IQR = 12.0). The median scores of mistrust of vaccine benefits, worries about unforeseen future effects, concerns about commercial profiteering, and preference for natural immunity were 6.0 (IQR = 3.0), 10.0 (IQR = 4.0), 6.0 (IQR = 4.0), and 7.5 (IQR = 4.0), respectively.

### 3.2. Factors Associated with Pneumococcal Vaccination Intention

The results of the univariate and multivariable logistic regression analyses for factors associated with pneumococcal vaccination intention are presented in Table 2. After controlling for background variables, age was negatively associated with pneumococcal vaccination intention (AOR = 0.956 [0.924, 0.990], *p* < 0.05). Participants with a household income greater than CNY 3000 were less likely to intend to receive the pneumococcal vaccine (AOR = 0.587 [0.388, 0.889], *p* < 0.05), while those living alone also exhibited a lower level of pneumococcal vaccination intention (AOR = 0.270 [0.103, 0.708], *p* < 0.01).

Participants with a history of influenza vaccination were more likely to intend to receive the pneumococcal vaccine (AOR = 1.675 [1.110, 2.527], *p* < 0.05). In terms of the negative attitudes toward general vaccination, mistrust of vaccine benefits (AOR = 0.906 [0.828, 0.990], *p* < 0.05) and concerns about commercial profiteering (AOR = 0.715 [0.631, 0.809], *p* < 0.001) were significantly associated with a lower level of pneumococcal vaccination intention.

### 3.3. Spearman Correlations of the Main Studied Variables

The Kolmogorov–Smirnov test revealed that the main studied variables did not follow a normal distribution. Consequently, the non-parametric method was applied, and the Spearman correlation was used for the analysis. Spearman correlation analysis revealed a significant positive correlation between influenza vaccination history and pneumococcal vaccination intention (r = 0.167, *p* < 0.001). In contrast, mistrust of vaccine benefits (r = −0.253, *p* < 0.001), worries about unforeseen future effects (r = −0.180, *p* < 0.001), concerns about commercial profiteering (r = −0.360, *p* < 0.001), and a preference for natural immunity (r = −0.212, *p* < 0.001) were negatively associated with pneumococcal vaccination intention (Table 3).

### 3.4. Mediations Between the History of Influenza Vaccination and Pneumococcal Vaccination Intention

**Figure 2:**
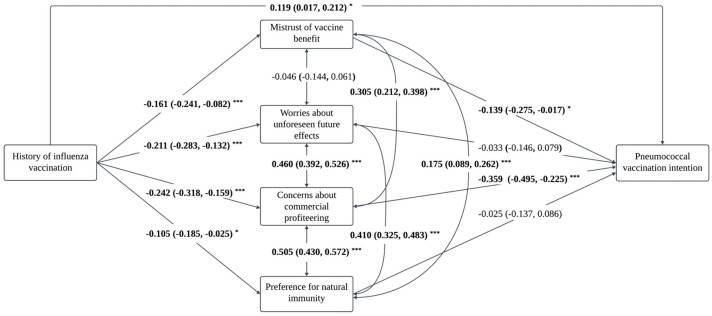
The proposed mediation model with standard coefficients for the pneumococcal vaccination intention (N = 658). *Note:* * *p* < 0.05, *** *p* < 0.001.

The results of the path analysis are presented in Figure 2 and Table 4. In the path analysis, statistical significance was detected in the total effect of influenza vaccination history on pneumococcal vaccination intention (standardized estimate = 0.238 [0.138, 0.329], *p* < 0.001), and the overall indirect effect mediated by negative attitudes toward general vaccination (standardized estimate = 0.119 [0.077, 0.168], *p* < 0.001). The indirect effect accounted for 50.0% of the total effect. The Comparative Fit Index (CFI), Tucker–Lewis Index (TLI), and Root Mean Square Error of Approximation (RMSEA) of this model were 0.947, 0.911, and 0.037, respectively, indicating a good model fit.

A total of two significant indirect paths were identified. Among the subscales of negative attitudes, concerns about commercial profiteering had the largest mediating effect (36.6%, standardized estimate = 0.087 [0.049, 0.136]), followed by mistrust of vaccine benefits (9.2%, standardized estimate = 0.022 [0.004, 0.054]). Worries about unforeseen future effects and preference for natural immunity did not significantly mediate the association between influenza vaccination history and pneumococcal vaccination intention.

## 4. Discussion

Attitudes toward vaccines influence vaccination behavior, especially vaccine hesitancy, which has been recognized as a global health threat by the WHO [32,33,34]. It is crucial to not only address the negative attitudes toward specific vaccines but also to understand the potential spillover effects between different vaccinations [35]. Based on the Behavioral Spillover Theory, this study explored the association between influenza vaccination history and pneumococcal vaccination intention, as well as the mediating role of negative attitudes toward general vaccination among older adults in China. Results showed that influenza vaccination history may reduce negative attitudes toward general vaccination, which may further increase pneumococcal vaccination intention, indicating spillover effects of influenza vaccination history. The study findings seem to provide valuable insights into the spillover effects between different vaccinations, offering recommendations for addressing anti-vaccine attitudes and vaccine hesitancy.

In this study, negative attitudes toward general vaccination may influence pneumococcal vaccination intention. After adjusting for background variables, the significant negative associations of pneumococcal vaccination intention with mistrust of vaccine benefits and concerns about commercial profiteering were observed. The finding was consistent with previous studies which found that willingness to vaccinate against pneumococcal was associated with confidence in vaccine effectiveness and concerns about profiteering [13,15,36]. This skepticism regarding the benefits may reduce participants’ motivation to receive the vaccine, as they may believe that the potential risks or side effects of vaccines outweigh their benefits [13]. Additionally, concerns about commercial profiteering may prompt participants to consider the pneumococcal vaccine as unnecessary or part of a profit-driven agenda, potentially resulting in their refusal to receive it [15]. Therefore, to promote vaccination uptake among older adults, addressing the relevant negative attitudes is essential and tailored strategies could be employed. Providing transparent communication about the benefits and effectiveness of vaccines along with collaboration with trusted healthcare providers could be considered, which may help mitigate older adults’ negative attitudes toward general vaccination and address vaccine hesitancy.

Influenza vaccination history may influence pneumococcal vaccination intention. This result is consistent with previous studies, which found that influenza vaccination increased the likelihood of receiving pneumococcal vaccination, indicating the spillover effects of influenza vaccination history [19,20,23]. Based on the Behavioral Spillover Theory [17,18], one possible explanation is that individuals who received the influenza vaccine may be more health-conscious and have a higher level of vaccine literacy, resulting in a higher likelihood of receiving other vaccines (e.g., pneumococcal vaccine) to protect themselves [37,38]. Since pneumococcal and influenza vaccines can be co-administered, one possible way of improving overall vaccination coverage is to enhance older adults’ vaccine literacy and encourage them to receive recommended vaccinations (e.g., pneumococcal vaccines) when they receive their flu vaccination [20,23,37].

Moreover, the negative attitudes toward general vaccination may play a mediation role in the association between influenza vaccination history and pneumococcal vaccination intention, as shown by the mediation analysis in the study. It is plausible that a prior unsatisfactory influenza vaccination experience increases individuals’ negative attitudes toward general vaccination, which may in turn result in hesitancy toward pneumococcal vaccination [39]. Therefore, to improve vaccination uptake, creating a more positive and supportive environment for vaccination for older adults seems to be crucial. To achieve this, high-quality vaccination services and tailored communication strategies may be considered [38]. In this study, the total indirect effect of negative attitudes toward general vaccination accounted for over 50.0% of the total effect of influenza vaccination history on the behavioral intention of receiving pneumococcal vaccination. However, the results of this study showed that worries about unforeseen future effects and preference for natural immunity were not significant mediators. To further examine the effects of these factors, future studies are needed.

Noteworthily, this study found that older adults living alone are less likely to receive the pneumococcal vaccine. This is consistent with previous studies conducted in Hong Kong, which found that living alone was negatively associated with pneumococcal vaccination behaviors among older adults [40]. This could be explained by the fact that older adults living alone may consider it unnecessary to be vaccinated because no others at their home need to be protected [32,41]. Similarly, this study found that age was negatively associated with pneumococcal vaccination intention, which aligns with prior literature studies [42]. One possible reason might be that oldest older adults may have greater concerns about complications after vaccination. Moreover, older adults with a higher income may be less likely to receive the pneumococcal vaccine. It may be because higher-income individuals have access to more information, including negative narratives about vaccines, which may increase their hesitancy. Therefore, when promoting vaccination among older adults, health professionals could pay more attention to those who live alone, are older, and have a higher income, providing them with additional community support, such as follow-up care after vaccination. It is highly likely that socio-cultural factors such as age, income, and education level vary across provinces. Older adults living in more developed provinces, such as Beijing, tend to have fewer negative attitudes toward vaccination compared to those in less developed provinces. However, this may be attributed to differences in socio-demographic variables, warranting future studies.

Based on the findings of this study, the observed spillover effects of influenza vaccination history on pneumococcal vaccination intention may give clues for improving adherence to vaccination. First, addressing common barriers (e.g., mistrust of vaccine benefits) is crucial. Public health campaigns could focus on providing comprehensive, evidence-based information about the benefits of routine vaccines (e.g., influenza and pneumococcal vaccines), targeting both the general population and specific high-risk groups (e.g., older adults). Second, efforts to promote and raise awareness about other vaccines can be strengthened at influenza vaccination sites. For instance, large screens at mass vaccination sites for the influenza vaccine could display promotional videos or information about other vaccines (e.g., pneumococcal vaccines). Last but not least, it is important to incorporate these two vaccines into routine healthcare visits and encourage healthcare providers to proactively discuss vaccines with older adults. For example, healthcare providers could recommend receiving the pneumococcal vaccination during the same visit as the influenza vaccination.

The study had some limitations. First, social desirability bias might influence the measurement of influenza vaccination history, negative attitudes toward vaccination, and behavioral intentions. The extent of the bias was difficult to gauge. To minimize the social desirability bias, this study used the self-administered questionnaire rather than the face-to-face interview. Participants who were unable to complete the electronic questionnaire independently would receive assistance from staff or family members, which may also result in social desirability bias. Second, this study used convenience sampling to recruit the participants, which may lead to selection bias. The influenza vaccination uptake rate may be overestimated, as older adults who are more familiar with community health services may be more inclined to participate in the study. Third, this study only evaluated the mediating role of four key domains of negative attitudes toward vaccination, including mistrust of vaccine benefits, concerns about unforeseen future effects, worries about commercial profiteering, and preference for natural immunity. Future studies are needed to explore other potential mediators (e.g., subjective norms) so as to gain a more comprehensive understanding of the mechanism between different vaccinations behaviors. Last but not least, causality could not be fully established in this study due to the cross-sectional design. Longitudinal studies are needed in the future.

## 5. Conclusions

In this study, a large-scale online survey was conducted among older adults in China, and the results demonstrated that influenza vaccination history may reduce negative attitudes toward general vaccination, which may further increase pneumococcal vaccination intention, indicating the spillover effects of influenza vaccination. These findings seem to indicate that addressing negative attitudes toward general vaccination among older adults is crucial. Therefore, to improve vaccination uptake among older adults in China and promote healthy aging in the future, targeted interventions and strategies are perhaps needed (e.g., providing high-quality vaccination services and collaborating with trusted healthcare providers) to enhance vaccine literacy and reduce anti-vaccine attitudes and vaccine hesitancy.

## Figures and Tables

**Table 1:** Distributions of all variables among older adults who did not receive the pneumococcal vaccination in China (N = 658).

Variables	n (%)/Median ± IQR						*p*-Value ^a^
	Total (n = 658)	Province A (n = 175)	Province B (n = 81)	Province C (n = 128)	Province D (n = 84)	Province E (n = 190)	
**Background variables**							
Age (years), Median ± IQR	65.0 ± 9.0	65.0 ± 11.0	63.0 ± 4.0	64.0 ± 6.0	64.0 ± 6.8	69.0 ± 11.0	<0.001
Gender							0.061
Male	308 (46.8)	70 (40.0)	32 (39.5)	62 (48.4)	46 (54.8)	98 (51.6)	
Female	350 (53.2)	105 (60.0)	49 (60.5)	66 (51.6)	38 (45.2)	92 (48.4)	
Education level							0.004
High school or below	607 (92.2)	157 (89.7)	78 (96.3)	112 (87.5)	75 (89.3)	185 (97.4)	
College and above	51 (7.8)	18 (10.3)	3 (3.7)	16 (12.5)	9 (10.7)	5 (2.6)	
Marital status							0.054
Married or living with a partner	569 (86.5)	151 (86.3)	78 (96.3)	112 (87.5)	70 (83.3)	158 (83.2)	
Unmarried/divorced/separated/widowed	89 (13.5)	24 (13.7)	3 (3.7)	16 (12.5)	14 (16.7)	32 (16.8)	
Employment status							<0.001
Retired	398 (60.5)	75 (42.9)	38 (46.9)	85 (66.4)	50 (59.5)	150 (78.9)	
Unemployed	224 (34.0)	87 (49.7)	42 (51.9)	37 (28.9)	30 (35.7)	28 (14.7)	
Employed	36 (5.5)	13 (7.4)	1 (1.2)	6 (4.7)	4 (4.8)	12 (6.3)	
Monthly household income (CNY)							<0.001
≤3000	290 (44.1)	103 (58.9)	49 (60.5)	58 (45.3)	29 (34.5)	51 (26.8)	
3001–5000	193 (29.3)	48 (27.4)	15 (18.5)	39 (30.5)	23 (27.4)	68 (35.8)	
>5000	175 (26.6)	24 (13.7)	17 (21.0)	31 (24.2)	32 (38.1)	71 (37.4)	
Living alone ^b^							0.384
Yes	61 (9.3)	22 (12.6)	7 (8.6)	10 (7.8)	9 (10.7)	13 (6.8)	
No	597 (90.7)	153 (87.4)	74 (91.4)	118 (92.2)	75 (89.3)	177 (93.2)	
Living with spouse	493 (74.9)	115 (65.7)	66 (81.5)	105 (82.0)	59 (70.2)	148 (77.9)	
Living with son/daughter	216 (32.8)	47 (26.9)	19 (23.5)	42 (32.8)	25 (29.8)	83 (43.7)	
Living with other family	48 (7.3)	19 (10.9)	5 (6.2)	7 (5.5)	6 (7.1)	11 (5.8)	
Medical insurance status							<0.001
Yes	558 (84.8)	152 (86.9)	75 (92.6)	118 (92.2)	75 (89.3)	138 (72.6)	
No	92 (14.0)	20 (11.4)	4 (4.9)	9 (7.0)	8 (9.5)	51 (26.8)	
Unknown	8 (1.2)	3 (1.7)	2 (2.5)	1 (0.8)	1 (1.2)	1 (0.5)	
Chronic disease status							0.006
Yes	517 (78.6)	140 (80.0)	64 (79.0)	93 (72.7)	57 (67.9)	163 (85.8)	
One chronic disease	273 (41.5)	59 (33.7)	44 (54.3)	46 (35.9)	25 (29.8)	99 (52.1)	
Two or more chronic diseases	244 (37.1)	81 (46.3)	20 (24.7)	47 (36.7)	32 (38.1)	64 (33.7)	
No/unknown	141 (21.4)	35 (20.0)	17 (21.0)	35 (27.3)	27 (32.1)	27 (14.2)	
**Vaccination-related behavior, intention, and perceptions**							
History of influenza vaccination ^c^							<0.001
Yes	363 (55.2)	79 (45.1)	64 (79.0)	44 (34.4)	30 (35.7)	146 (76.8)	
No	295 (44.8)	96 (54.9)	17 (21.0)	84 (65.6)	54 (64.3)	44 (23.2)	
Whether heard of pneumococcal vaccination							<0.001
Yes	220 (33.4)	45 (25.7)	36 (44.4)	58 (45.3)	37 (44.0)	44 (23.2)	
No	438 (66.6)	130 (74.3)	45 (55.6)	70 (54.7)	47 (56.0)	146 (76.8)	
Pneumococcal vaccination intention							<0.001
Very unlikely	68 (10.3)	13 (7.4)	6 (7.4)	17 (13.3)	6 (7.1)	26 (13.7)	
Unlikely	111 (16.9)	28 (16.0)	7 (8.6)	26 (20.3)	9 (10.7)	41 (21.6)	
Half and half	295 (44.8)	89 (50.9)	25 (30.9)	53 (41.4)	44 (52.4)	84 (44.2)	
Likely	107 (16.3)	35 (20.0)	20 (24.7)	15 (11.7)	18 (21.4)	19 (10.0)	
Very likely	77 (11.7)	10 (5.7)	23 (28.4)	17 (13.3)	7 (8.3)	20 (10.5)	
Negative attitudes toward general vaccination (range: 12–52), Median ± IQR	28.5 ± 12.0	29.0 ± 10.0	24.0 ± 14.5	30.5 ± 9.0	31.5 ± 8.0	26.0 ± 11.3	<0.001
Mistrust of vaccine benefit (range: 3–15)	6.0 ± 3.0	6.0 ± 3.0	3.0 ± 3.0	6.0 ± 3.0	6.0 ± 3.0	6.0 ± 3.0	<0.001
Worries about unforeseen future effects (range: 3–15)	10.0 ± 4.0	10.0 ± 4.0	9.0 ± 3.0	10.5 ± 4.0	11.0 ± 2.8	8.0 ± 3.0	<0.001
Concerns about commercial profiteering (range: 3–15)	6.0 ± 4.0	6.0 ± 4.0	4.0 ± 3.0	6.0 ± 5.0	6.0 ± 3.0	5.0 ± 3.0	<0.001
Preference for natural immunity (range: 3–11)	7.5 ± 4.0	8.0 ± 3.0	6.0 ± 5.0	8.0 ± 5.8	9.0 ± 2.0	7.0 ± 4.0	<0.001

*Note:* IQR, interquartile range. CNY, Chinese yuan. An exchange rate of CNY 1 = USD 0.14 was applied. ^a^ To compare the differences in background variables, vaccination-related behaviors, intentions, and perceptions across different regions, chi-square tests or Fisher’s exact tests were used for categorical data, while Kruskal–Wallis H tests were applied to continuous data that did not follow normal distribution. ^b^ Participants could choose multiple choices simultaneously. ^c^ Participants were asked to report their history of influenza vaccination in the past 12 months.

**Table 2:** Factors associated with pneumococcal vaccination intention among older adults who did not receive the pneumococcal vaccination in China (N = 658).

Variables	Pneumococcal Vaccination Intention	
	OR (95%CI) ^a^	AOR (95%CI) ^b^
Background variables		
Age (years)	0.972 (0.946, 0.999) *	0.956 (0.924, 0.990) *
Gender		
Male	Ref	Ref
Female	1.542 (1.091, 2.181) *	1.389 (0.938, 2.055)
Education level		
High school or below	Ref	Ref
College and above	0.872 (0.453, 1.678)	1.322 (0.622, 2.810)
Marital status		
Unmarried/divorced/separated/widowed	Ref	Ref
Married or living with a partner	1.300 (0.771, 2.191)	0.841 (0.421, 1.678)
Employment status		
Unemployed	Ref	Ref
Employed/retired (with pension)	1.109 (0.784, 1.569)	1.215 (0.785, 1.882)
Monthly household income (CNY)		
≤3000	Ref	Ref
>3000	0.675 (0.480, 0.950) *	0.587 (0.388, 0.889) *
Living alone		
No	Ref	Ref
Yes	0.308 (0.137, 0.689) **	0.270 (0.103, 0.708) **
Medical insurance		
No/unknown	Ref	Ref
Yes	1.059 (0.656, 1.708)	1.393 (0.804, 2.414)
Chronic disease		
No/unknown	Ref	Ref
Yes	0.752 (0.503, 1.125)	0.945 (0.589, 1.515)
**Vaccination-related behavior and perceptions**		
History of influenza vaccination		
No	Ref	Ref
Yes	2.172 (1.517, 3.112) ***	1.675 (1.110, 2.527) *
Negative attitudes toward general vaccination		
Mistrust of vaccine benefit	0.787 (0.720, 0.860) ***	0.906 (0.828, 0.990) *
Worries about unforeseen future effects	0.847 (0.791, 0.908) ***	0.977 (0.895, 1.065)
Concerns about commercial profiteering	0.673 (0.612, 0.741) ***	0.715 (0.631, 0.809) ***
Preference for natural immunity	0.814 (0.748, 0.887) ***	0.967 (0.901, 1.039)

*Note:* * *p* < 0.05, ** *p* < 0.01, *** *p* < 0.001. OR, odds ratio. CI, confidence interval. ^a^ Univariate logistic regression was performed for each independent variable. ^b^ Multivariable logistic regression was performed, including for all the independent variables. The Hosmer–Lemeshow Test showed that χ^2^ was 10.463 and the *p*-value was 0.234, suggesting that the logistic regression model fits the data well. The Nagelkerke R^2^ value was 0.257, indicating that 25.7% of the variation in the outcome variable is explained by the predictors in the model.

**Table 3:** Spearman correlations of the main studied variables among older adults who did not receive the pneumococcal vaccination in China (N = 658).

Variables	1	2	3	4	5
1. History of influenza vaccination	-				
2. Pneumococcal vaccination intention	0.167 ***	-			
3. Mistrust of vaccine benefit	−0.210 ***	−0.253 ***	-		
4. Worries about unforeseen future effects	−0.236 ***	−0.180 ***	0.131 ***	-	
5. Concerns about commercial profiteering	−0.262 ***	−0.360 ***	0.477 ***	0.514 ***	-
6. Preference for natural immunity	−0.118 **	−0.212 ***	0.305 ***	0.433 ***	0.519 ***

*Note:* ** *p* < 0.01, *** *p* < 0.001.

**Table 4:** Path analysis of the mediation effect of negative attitudes toward general vaccination on the association between history of influenza vaccination and pneumococcal vaccination intention among older adults who did not receive pneumococcal vaccination in China (N = 658).

Paths	Standardized Estimate	95% CI	% of the Total Effect
**Outcome: pneumococcal vaccination intention**			
**1. Total effect**	0.238	(0.138, 0.329)	\
**2. Direct effect**	0.119	(0.017, 0.212)	50.0%
**3. Indirect effect**	0.119	(0.077, 0.168)	50.0%
(1) History of influenza vaccination → mistrust of vaccine benefit → outcome	0.022	(0.004, 0.054)	9.2%
(2) History of influenza vaccination → worries about unforeseen future effects → outcome	0.007	(−0.017, 0.033)	2.9%
(3) History of influenza vaccination → concerns about commercial profiteering → outcome	0.087	(0.049, 0.136)	36.6%
(4) History of influenza vaccination → preference for natural immunity → outcome	0.003	(−0.008, 0.018)	1.3%

*Note:* The estimation was based on 5000 bootstrapped samples; CI, confidence interval.

## Data Availability

The data presented in this study are available from the corresponding author upon request. The data are not publicly available as they contain personal behaviors.
